# Text-Book of Ophthalmology

**Published:** 1893-02

**Authors:** 


TEXT-BOOK OF OPHTHALMOLOGY. By DR. ERNEST FUCHS, Professor of Ophthalmology in the University of Vienna. Authorized translation from the second enlarged and improved German edition, by A. DUANE, M. D., Assistant Surgeon Ophthalmic and Aural Institute, New York. With numerous illustrations. Octavo, pp. 788. Price, $5.00. Sold by subscription. New York: D. Appleton & Co. 1892.

The multiplicity of text-books on diseases of the eye would seem, at the present time, to be sufficient to supply the demand for works of this kind, and contra-indicate the issue of any more, at least for a while, particularly when those already published possess such intrinsic merit as is recognized in Noyes, Swanzy, Berry, Schmidt-Kimpler, Meyer, Juler, de Schweinitz, and others. But when such a man as Fuchs offers the results of his vast experience to the profession, he is sure to find a most generous welcome, no matter who has gone before him. From the first appearance of Fuchs's text-book in German, it has received high praise, and has been regarded as the work of a master hand. The English-speaking part of the profession has desired to see it in familiar vernacular, and has impatiently awaited its translation. The publishers did not misjudge public sentiment when they assumed that there was a demand for this particular treatise. Dr. A. Duane, of New York, has taken upon himself the task of rendering it into English, and has so well succeeded that it is very easy and pleasant reading. Not only is the book put into good English, but the translator has, in his efforts to Americanize it, made some additions which materially enhance its value. These, however, he has separated from the text by brackets, thus assuming entire responsibility for them.

The work is designed especially as a "text-book," and as such is supposed to contain simply the "essentials" of ophthalmology.

From reading it, however, one is led to feel that the author's idea of "essentials" is more extended than that of most text-book writers, as so much is to be found here that one would look for in vain in other works of the kind. Indeed, into its pages is compressed a vast amount of information bearing upon pathology and practice, and much of it is the outcome of the author's large and mature experience. Thus the work becomes of value to the experienced specialist as well as to the student.

The subjects are divided so that part first includes examination of the eye; part second, diseases of the eye, including those of the structures of the ball, the conjunctiva, lachrymal organs, lids, muscles, and nerves, and orbit; and part third, anomalies of refraction and operations. The strength of the book lies in part second. Here the diseases of the conjunctiva, cornea, sclera, iris, ciliary body, "chorioid," lens, vitreous, retina, optic nerve, lids, lachrymal organs, orbits, and glaucoma are treated most fully. Suitable anatomical descriptions introduce the several chapters, and in the chapter on the Anatomy and Physiology of the Uvea is included the Embryology of the Eye. This subject may seem to be somewhat misplaced, but this does not detract from its own value or interest, or that of the excellent chapter into which it is incorporated. As suggestive of the exhaustive character of this second part of the work, the chapter on Diseases of the Conjunctiva occupies ninety pages; that on Diseases of the Cornea, eighty-nine pages; that on the Anatomy and Diseases of the Iris, Ciliary Body, and "Chorioid," ninety-six pages; that on the Optic Nerve, thirty-three pages; and that on the Lids, forty-eight pages.

Throughout the work, the author has been careful to make the spelling of words correspond to their proper etymology. For example, for "myosis" he writes "miosis," and for "choroid" he writes "chorioid." There is no doubt about the author being etymologically correct. "Hemeralopia" is used to mean night-blindness (day-sight), and "nyctalopia" day-blindness (night-sight). This is the reverse of that recognized by many English ophthalmologists and some American. In 1881 and 1882, Dr. W. A. Greenhill and Mr. John Tweedy (*Royal London Ophthalmic Hospital Reports*, Vol. X., pp. 284 and 413,) examined most carefully into the original meaning of the derivatives of these words, and concluded that "nyctàlopia" means night-blindness, and "hemeralopia" day-blindness. Their conclusions have been accepted by many. In the present confusion, however, it might be better to omit the Greek terms and use only the English, "night-blindness" and "day-blindness," as several have already done.

The weakness of the work is in part third, and in the chapter on Disturbances of Motility, in part second. The subjects of these are disposed of much more summarily. Much that is today esteemed of value in the refraction and "muscularity" of the eye by Americans is entirely ignored by the author, or, if not ignored, is somewhat at variance with views held by them. For example, we fail to find the subject of convergence of the eyes treated of in the work, and, therefore, no mention is made of Nagel's system of "metre-angles," which is now recognized everywhere in scientific ophthalmology, or of the various muscle-tests now in vogue. The American ophthalmologist will also criticise the author's views on the correction of refractive errors, and his non-recognition of the relation of muscular and refractive anomalies of the eyes to headaches and other neuroses.

Notwithstanding what may be regarded by some as a great defect in part third, the other chapters are so good that the work, as a whole, is to be commended to the student and specialist, and may be sought as a safe and trustworthy guide, so far as it goes. Americans must not expect the most light to come from the Germans on so-called "functional disorders" of the eye; but in "organic" diseases of this organ, by their patient, painstaking, and exact researches, they lead the world. In these, too, Fuchs becomes master. A. A. H.

